# Onion thrips (*Thrips tabaci* (Thysanoptera: Thripidae)) in cabbage on Prince Edward Island: observations on planting date and variety choice

**DOI:** 10.1186/s40064-015-1221-2

**Published:** 2015-08-19

**Authors:** Suzanne Blatt, Andrew Ryan, Shelley Adams, Joanne Driscoll

**Affiliations:** Agriculture and Agri-Food Canada, 32 Main Street, Kentville, NS B4N 1J5 Canada; PEI Horticultural Association, 420 University Avenue, Charlottetown, PE C1A 7Z5 Canada

**Keywords:** Onion thrips, Cabbage, Prince Edward Island, Variety preference, Planting time

## Abstract

Onion thrips (*Thrips tabaci* Lindeman (Thysanoptera: Thripidae)) can be a pest in organic onion production on Prince Edward Island. This study was to examine the effect of planting time and variety on infestation levels and damage by onion thrips on cabbage (*Brassicae oleraceacapitala* (L.)). A field site was planted with 2 main and 8 lesser varieties of cabbage over 4 planting dates. Some varieties were short season and harvested on July 31 with longer season varieties harvested on September 2. Blue sticky traps were used to capture thrips migrating into the field site from July 22–September 2. Traps were counted weekly and cabbage heads within the field site were visually surveyed for thrips. At harvest, heads were weighed and measured, thrips damage was assessed then the head was dissected and thrips counted on the first four layers of the head. Thrips exhibited a preference for Lennox over Bronco throughout the season although thrips populations were not high enough to effect economic damage in 2014. Planting date influenced cabbage head weight and size with later plantings yielding the largest heads. Use of planting date and variety to avoid thrips populations is discussed.

## Background

Onion thrips represent a serious threat to cabbage production in cabbage growing regions of the world (Garamvölgyi et al. 2004; Shelton et al. 2008). Damage can appear as a bronze discoloration and/or a rough texture on leaves within the head (North and Shelton 1986b) causing injury up to 20 layers deep (Trdan et al. 2005; Respondek and Zvalo 2008) and reducing marketable production. For Prince Edward Island (PEI), thrips can cause significant loss to the cabbage crop in organic fields. Control is often recommended at the early head formation stage (7.5 cm leaf ball) (PEI Department of Agriculture, Fisheries and Aquaculture 2005), with pesticides available for conventional producers (Shelton et al. 1998; Trdan et al. 2007) but no viable products available for organic production. The decision to spray is currently based on a presence/absence determination of thrips. Avoiding thrips populations through selection of planting date and less susceptible varieties are two potential options available to organic growers. Varietal preference and susceptibility or resistance to thrips has been documented in Europe and the United States. Some of the traits investigated to explain this preference include leaf wax thickness, reflectance or color of the leaves around the head. In Hungary, 52 white cabbage varieties were evaluated for resistance to thrips damage. None of the varieties was completely resistant although a handful of varieties showed decreased susceptibility (Garamvölgyi et al. 2004). Other studies have found *Thrips tabaci* to exhibit a variety preference using locally available commercial varieties (Trdan et al. 2005; Shelton et al. 1998). Shelton et al. (2008) found planting date and variety to significantly influence the extent of thrips damage in the top 10 layers of cabbage head leaves. Cabbage planted later in the season (July 25 vs. July 3) experienced less damage for specific varieties. It is speculated that these later planted cabbage heads experienced lower thrips pressure due to reduced immigration into the field (North and Shelton 1986b; Shelton and North 1986), or increased mortality due to high rainfall during the latter part of the season. Of the varieties commonly planted in PEI, it is unknown whether thrips show a preference, or if selecting early developing varieties and planting early in the season will serve to avoid the thrips populations.

The objectives of this work were to evaluate the influence of planting date and cultivar on the incidence of *T. tabaci* within a commercial organic cabbage planting, and determine if monitoring with sticky traps can predict thrips damage.

## Results

**Fig. 1:**
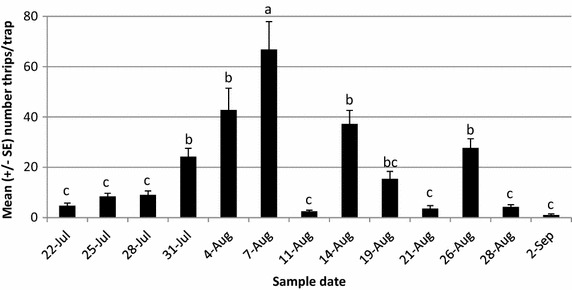
Mean ± SE number of thrips captured on 17 blue sticky traps (all locations pooled for each date) around a cabbage field on PEI in 2014. Trap catches showed significant differences by date (*P* < 0.001). *Bars* with the *same letter* denote no significant difference

**Fig. 2:**
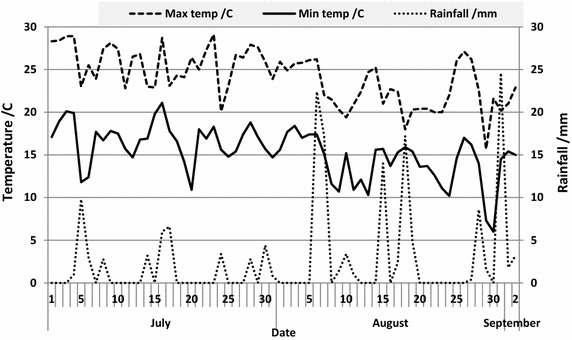
Daily maximum and minimum temperatures and rainfall over the sampling period in 2014

In total 3996 thrips were captured on 17 traps surrounding the study plot. The majority of the population was caught by August 7 (63 %). Thrips trap catches over the growing season showed a gradual increase, then decrease as the cabbages ripened (Fig. 1), significant across trapping dates (F_12,196_ = 19.3, *P* = 2.0 × 10^−16^). One date stands out as an anomaly in terms of trap catch, August 11. On this date numbers are lower than expected given the captures before and after that date. It is possible this occurred due to increased rainfall on August 6 and 7 (Fig. 2). Rainfall was again high between August 15 and 18, which could explain the lower trap catches on August 19 and 21. A negative influence of heavy rainfall on thrips populations was observed by Leite et al. (2005, 2006) in Slovenia and again by Morsello et al. (2008) in North Carolina and Virginia, USA.

**Fig. 3:**
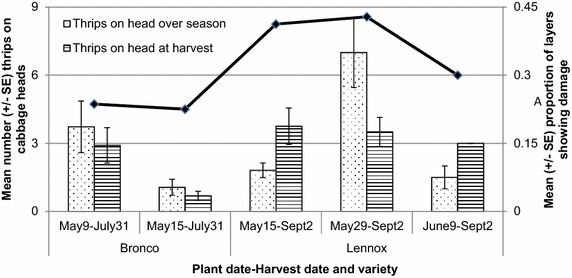
Mean (±SE) of thrips observed on cabbage heads over the growing season by planting date-harvest date and variety

Location of the traps around the field site strongly influenced capture numbers (Table 1). When examined by location, more thrips were captured on the eastern edge of the field over the north or southern edges. Highest catches were observed on the traps located 3 m outside the southern edge of the field. Thrips found on the cabbage heads throughout the growing season showed significant differences by planting date, harvest date and variety (Table 2; Fig. 3). Cabbages planted on May 29 experienced significantly more thrips than other planting dates. Not unexpectedly plants remaining in the field until the later harvest date experienced more thrips over the season than those harvested earlier. There was no linear relationship between number of growing days in the field and thrips found on the cabbage heads over the season (R^2^ = 0.002).

**Table 1:** Cumulated weekly trap counts, mean (SE), for thrips near a cabbage field in North Wiltshire, PEI, from July 22 to September 2, 2014 showing impact of location on thrips abundance throughout the season

Week^+^	Trap location				F(df), *P*
	North	East	South-edge	South-outer*	
1	1.0 (0.0)	5.6 (1.9)	3.0 (2.0)	7.3 (1.9)	1.5 (3,11), 0.26
2	8.0 (2.5)^b^	25.4 (4.8)^a^	15.0 (3.6)^ab^	19.3 (1.7)^ab^	4.2 (3,13), 0.03
3	24.0 (4.6)^c^	72.2 (14.6)^b^	39.0 (6.5)^bc^	121.0 (13.6)^a^	13.2 (3,13), 3.05 × 10^−4^
4	39.5 (11.6)^b^	60.3 (7.8)^b^	37.7 (3.7)^b^	140.0 (4.7)^a^	37.4 (3,12), 2.28 × 10^−6^
5	14.7 (1.2)^c^	53.3 (4.6)^b^	49.0 (7.6)^b^	93.5 (6.9)^a^	31.1 (3,12), 6.10 × 10^−6^
6	7.7 (1.5)^b^	42.0 (2.7)^a^	36.7 (2.0)^a^	38.5 (6.8)^a^	16.2 (3,12), 1.62 × 10^−4^
7	1.5 (0.6)^b^	6.7 (1.7)^b^	3.5 (1.2)^b^	8.3 (1.6)^a^	4.52 (3,12), 0.02
All weeks	14.7 (3.0)^c^	37.6 (5.0)^b^	26.3 (4.0)^bc^	61.1 (10.0)^a^	9.7 (3109), 1.03 × 10^−5^

Letters within each week denote significant differences using Tukeys’ HSD

* traps placed 3 m from edge of the field

^+^Week 1: July 21–24, Week 2: July 25–28, Week 3: Jul 31–Aug 4, Week 4: Aug 7–11, Week 5: Aug 14–19, Week 6: Aug 21–26, Week 7: Aug 28–Sept 2

**Table 2:** ANOVA table for number of thrips found on cabbage heads during season and at harvest and proportion of layers showing damage for Bronco and Lennox varieties

Variable	Variety (F_df_, *P*)	Plant date (F_df_, *P*)	Harvest date (F_df_, *P*)
Thrips on heads over season	1.77_1,55_, 0.19	12.31_3,55_, 2.86 × 10^−6^	33.41_1,55_, 3.64 × 10^−7^
Thrips on heads at harvest	7.13_1,55_, 9.99 × 10^−3^	2.27_3,55_, 8.99 × 10^−2^	10.75_1,55_, 1.82 × 10^−3^
Proportion of layers showing damage	5.92_1,55_, 1.82 × 10^−2^	0.12_3,55_, 0.95	1.65_1,55_, 0.20

**Fig. 4:**
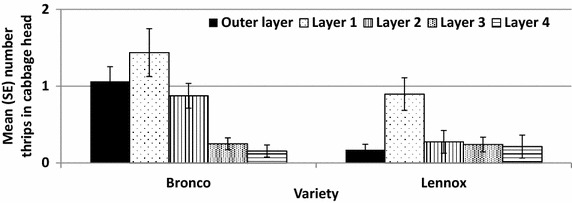
Mean number (±SE) of thrips on Bronco and Lennox cabbage heads at harvest by location. Variety was significant for the outer layer and layer 2 only (*P* < 0.001). Other layers were not significant for variety, planting date or harvest date (*P* > *0.05*). Varieties not shown were excluded from the analysis due to lack of replication

Thrips captured on the traps over the season showed no correlation with thrips observed on the cabbage heads (R^2^ = −0.003). Thrips observed during last cabbage head survey (immediately before harvest) were correlated with thrips observed on the head during the damage evaluation (r = 0.28, n = 69, *p* = 0.017). Evaluated by harvest date, the correlation was higher, and significant, for cabbages harvested early (r = 0.48, n = 30, *p* = 0.0069) compared with cabbages harvested late (r = 0.11, n = 39, p = 0.51). Cabbages were dissected after harvest and variety was found to significantly influence the location of thrips within the cabbage head (F_1,55_ = 18.6, *P* = 6.7 × 10^−5^ and F_1,55_ = 13.9, *P* = 4.6 × 10^−4^, outer layer and layer 2, respectively, Fig. 4) while planting date and harvest date did not (*P* ranged from 0.05 to 0.53 and 0.11 to 0.68, plant date and harvest date, respectively). Thrips were more likely to be found in the 1st layer of the cabbage head in Bronco and Lennox. Over all layers, Lennox harbored more thrips than Bronco (3.78 and 1.8 thrips per head, Lennox and Bronco, respectively) although these two varieties harbored lower numbers compared with some of the other varieties, e.g. Bruno, CLX, Prime Vantage and Tiara. Location of the thrips within the cabbage head varied by variety with some harboring greater numbers in the first layer (Bronco, Grand Vantage and Tiara) while the second layer harbored greater thrips numbers for BC63, Bruno, CLX and Prime Vantage. Replication within these other varieties was too low for statistical analysis, so these observations must be considered anecdotal. These findings differ from other studies which show thrips to occur on the first 17 leaves of the cabbage head (North and Shelton 1986a).

**Fig. 5:**
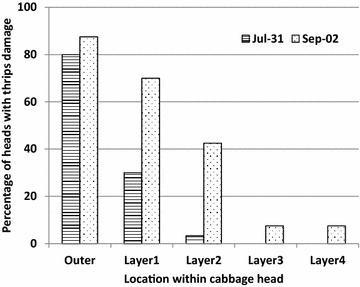
Percentage of cabbage heads, at harvest, showing thrips damage by harvest date. Location within the head and harvest date were significant (*P* = 0.02)

A higher percentage of heads exhibited damage more prominently on the outer and 1st layers of the head than on subsequent layers (χ^2^ = 11.89, *P* = 0.02, Fig. 5). This finding is consistent with results found by Trdan et al. 2007. Proportion of layers damaged within the head did not correlate well with the number of thrips found on the heads at harvest, nor the number of thrips found on the heads throughout the season (r = 0.43, n = 70, *p* < 0.001 and r = 0.38, n = 70, *p* = 0.001, thrips at harvest and thrips over the season, respectively) for both harvest dates. When considered by harvest date, proportion of layers damaged did not correlate well with the number of thrips found on the heads at harvest (r = −0.16, n = 30, *p* = 0.41), whereas the correlation was significant for the later harvest date (r = 0.6, n = 40, *p* < 0.0001). Proportion of the cabbage head experiencing damage was not correlated with thrips found on the heads over the season for the early planting (r = 0, n = 30, *p* = 0.98) but was significant for the later harvest date (r = 0.38, n = 40, *p* = 0.02). A stronger correlation for cabbages harvested later is consistent with the thrips having a longer time period to establish and colonize the heads.

**Fig. 6:**
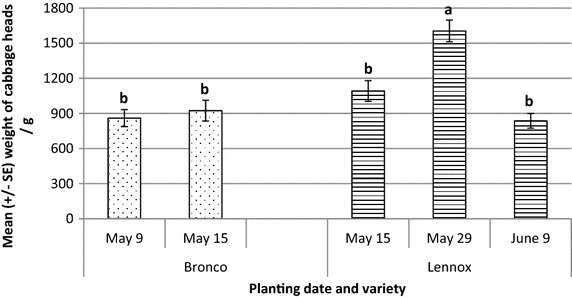
Head weight (in grams) of cabbage at harvest (September 2) by cultivar and planting date from thrips study on PEI in 2014. Bronco was significantly heavier than Lennox at harvest (*P* < 0.001), with the heaviest heads resulting from the May 29 planting

**Fig. 7:**
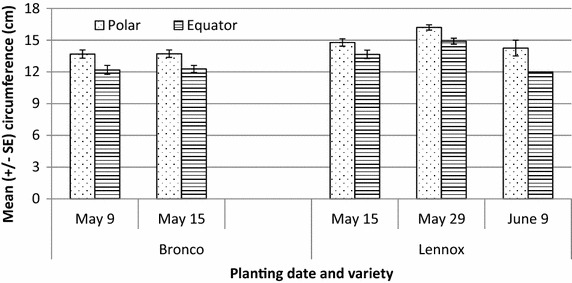
Dimensions of cabbage at harvest taken along the polar and equator axes of the head by cultivar and planting date from thrips study on PEI in 2014. Lennox was significantly larger than Bronco along both axes (*P* = 0.001) with the May 29 planting date yielding the largest heads

Head weight and size was evaluated for Lennox and Bronco. Lennox produced heavier heads when planted later (May 29, F_3,54_ = 7.6, *P* = 2.6 × 10^−4^) and these heads were heavier than Bronco planted on either May 9 or May 15 (F_1,54_ = 22.0, *P* = 1.8 × 10^−5^, Fig. 6; Table 3). In addition, Lennox cabbage heads were larger in both dimensions over Bronco heads, with the later planting of Lennox producing the largest heads (Fig. 7). Lennox heads were, on average, 10 % larger along both polar and equatorial dimensions (F_1,55_ = 26.2, *P* = 4.1 × 10^−6^ and F_1,55_ = 28.7 and *P* = 1.7 × 10^−6^, polar and equator, respectively). Planting date did not affect final head size for Bronco cabbage heads. Planting date for Lennox did affect final head size for Lennox. Cabbages planted on May 29 were 9 % larger than heads planted on May 15 and 18 % larger than those planted on June 9. This suggests that planting a bit later in the season allows plants to avoid the cooler early season temperatures which could delay their development.

**Table 3:** ANOVA table for cabbage weight and size showing significance of variety and planting date, F (*P* value)

Variable	Variety_1,54_	Plant date_3,54_
Head weight	22.07 (1.85 × 10^−5^)	7.56 (2.61 × 10^−4^)
Polar circumference	26.18 (4.08 × 10^−6^)	3.78 (1.54 × 10^−2^)
Equator circumference	28.72 (1.70 × 10^−6^)	3.94 (1.28 × 10^−2^)

## Discussion

*Thrips tabaci* were readily captured on unbaited sticky traps throughout the growing season. The population moved from surrounding vegetation into the crop and was more abundant on the south and eastern sides of the plot. This was unexpected as the east side of the field site contained the main roadway access to the field and therefore was considered non-favorable to thrips survival. Weed and plant species present around the north, east and south sides of the field did not differ markedly in composition containing a mixture of *Plantago* spp., *Poa* spp., *Taraxacum* spp., *Aster* spp., *Erigeron* spp. and *Lactuca* spp. Plants along the eastern side of the field were frequently crushed by tractors, cultivators and other farm equipment. Given the lower plant quality and the frequent disturbances, higher thrips along this side seems counter-intuitive. Along the south edge of the field, traps located 3 m off the field edge had the highest captures. To the south of these traps, the adjacent field contained fall rye. It is conceivable that cabbage is more attractive to *T. tabaci* than fall rye and this caused a general migration away from that field (planted in lettuce in 2013) and into the cabbage field. Thrips are known to use lettuce as a host (Workman et al. 2007) and could have built up populations during 2013, then moved towards the cabbage in 2014. A similar argument could exist for the field across the roadway which was also planted in fall rye during 2013. Rye has been noted as a host for *T. tabasci* but is not recorded as a major pest of this crop. It is quite likely that the planting scheme chosen focused the thrips to move toward the cabbage planting in 2014. Additionally, consideration of the general wind direction may have contributed to the pattern of trap catches observed. From July 28 through August 14, the bulk of the population was captured on sticky traps located along the south and east edges (facing away from the cabbage field). During that time, according to Environment Canada records, the wind direction was predominantly from the North–East and South which would have blown the thrips onto the South and East traps more so than those located on the North edge of the cabbage plot. It is clear that distribution within the field is influenced by factors other than those examined during this study. A broader examination of the landscape and correlation with thrips number may provide insight into the likelihood of thrips survival in natural habitats. Temperature was found to be the main factor influencing thrips populations (Morsello et al. 2008). In this study the population gradually increased then decreased by early September coinciding with both a decrease in daily temperatures and shorter day length. Should climate change result in warmer temperatures for this region, then the population could have multiple generations in a year (Bergant et al. 2005) creating the potential for damage later in the season. A further factor to consider is haplotype. Nault et al. 2014 found haplotype populations of thrips on cabbage to change significantly during the growing season in New York during 2005 and 2007. It is known that different haplotypes have different reproductive rates and ability to transmit viruses. It is therefore possible that if the haplotypes in cabbage in PEI change, that this could affect the extent of damage observed on cabbage towards the end of the growing season.

Despite the large numbers of thrips captured on the traps, thrips observed on the cabbage heads over the season were very low. This suggests that either the thrips were not able to effectively colonize the heads in the field or that visual surveys were unable to adequately detect a representative portion of the population present on the head. Correlation between thrips observed during the last cabbage head survey did not correlate well with thrips found during cabbage head damage evaluation for cabbages harvested later in the season. The correlation was higher for cabbage heads harvested earlier. This suggests that head surveys are not indicative of the population contained within the head once the bulk of the population has moved into the field. Alternative means to quantify the thrips present in the plot itself will need to be evaluated. Damage from thrips was negligible at harvest and none of the observed damage was significant enough to render the cabbage heads unmarketable.

*Thrips tabaci* showed a varietal preference for Lennox over Bronco throughout this study. The variety Bronco is considered an earlier variety requiring fewer days to harvest than Lennox. In this study, Bronco was harvested after 76 and 82 days (May 15 and May 9 planting dates, respectively). This is longer than the stated days to harvest of 64–68 days indicated for this variety. In North Dakota, Greenland et al. (2000) found Bronco to require 116–131 days to mature, depending upon year. The same study found temperature to correlate with yield. Lennox was harvested after 83, 93 and 107 days in the field (June 9, May 29 and May 14, planting dates, respectively). This is shorter than the 105 days to maturity more typical in other areas.

Variety preference for thrips has been studied in cabbage for some time. While the varieties used in this study were rarely included in these studies, they could provide future directions for inquiry. Voorrips et al. (2008, 2010) evaluated accessions of cabbage known to be susceptible and resistant to thrips attack. Over the years of the study, there were several of the traits which explained the variation in damage observed, however Brix and leaf wax layer late in the season (just before harvest) were the two traits that were consistent over varieties and years. Trdan et al. 2004 studied 6 varieties and found wax to differ among varieties in accordance with thrips damage. Study of plant compounds in cabbages identified as resistant to *T. tabaci* showed no correlation with damage (Trdan et al. 2008a). The strongest factor influencing thrips damage was wax content of the cabbage leaves. Fail et al. (2008) studied light reflectance from cabbage leaves and found the outer head leaves to differ. The color of the outer head-forming leaves did correlate with the number of thrips adults found in the cabbage heads. A follow up study by Fail et al. (2013) demonstrated that light reflectance can correlate with thrips presence in some years but not others, suggesting that other variables may influence thrips’ response to spectral cues. Bálint et al. (2013a) evaluated epidermal thickness between resistant and susceptible cabbage varieties. Thickness did not differ between varieties and did not correlate with thrips damage. Bálint et al. (2013b) studied leaf reflectance of UV-A, and visible light wavelengths. Their findings confirmed a negative relationship between the UV-A light reflection and onion thrips during the early stages of cabbage head formation. It is suggested that more intensive reflection of leaves may deter onion thrips from establishing on these varieties. As this was the first examination of varietal preference of thrips for cabbage on Prince Edward Island, it was unclear if a preference would be observed. Future studies on Prince Edward Island should endeavour to investigate the mechanism of the varietal preference observed.

Earlier planting and harvest of Bronco did result in avoidance of the majority of the invading thrips. By July 31, the date of harvest for Bronco, only 20 % of the immigrating thrips were captured on the sticky traps. The remaining 80 % of the migrating thrips were captured over the month of August. Even with the bulk of the population occurring after the harvest of Bronco, thrips populations were not high enough to cause economic losses to cabbages harvested in September of 2014. Head weight and size was larger for the cultivar Lennox and the later planting, but no correlation between head weight and thrips presence on the cabbages was observed. Lennox cabbage heads at harvest (September 2) were largest from the later planting date of May 29. This differs from the literature where Kleinhenz and Wszelaki (2003) found planting date and cultivar to be significant for head weight and size with earlier plantings producing significantly heavier heads from later plantings. Bronco was one of these varieties and performed reasonably well with 4 of the 10 traits examined affected by planting date. These included weight, volume, and polar and equatorial dimensions.

Organic cabbage producers on Prince Edward Island have few options with respect to thrips management. Varietal resistance, selection of short season varieties and timing of planting have promise in managing the naturally occurring thrips populations. Other options that organic growers may explore in future include intercropping or mulching. Intercropping of cabbage with a clover (Teunissen et al. 1995) or encouraging clover in the grassways around the fields may serve to increase natural enemies (Pobožniak and Wiech 2005). Another option may be mulching which Trdan et al. (2008b) found to show potential for increasing early cabbage production. While not examined in this study, the reproductive modes of *T. tabaci* may offer an opportunity for control. Li et al. (2014) found thelytokous *T. tabaci* to be better adapted to survive on cabbage while arrhenotokous *T. tabaci* were better adapted for onion. These reproductive modes influence longevity and fecundity which may, in turn, provide opportunities when deciding pest management strategies.

## Conclusions

The cabbage variety Bronco was less preferred by thrips. The more preferred variety Lennox when planted and harvested earlier in the growing season harbored fewer thrips and experienced lower damage than Lennox planted and harvested later in the season. Planting varieties with tolerance to thrips damage may serve to protect cabbages without use of insecticides. For organic growers on PE Island, there are currently no available chemical products to protect their crop. Thrips have demonstrated a preference for certain cabbage varieties in other parts of the world and these have been used successfully to reduce thrips damage. Timing of planting and harvest may also play a role in avoiding damage from thrips. This study demonstrates that commercially available varieties planted and harvested early in the season on PE Island exhibit reduced damage from thrips.

## Methods

### Cabbage planting dates, cultivars and data collected

**Fig. 8:**
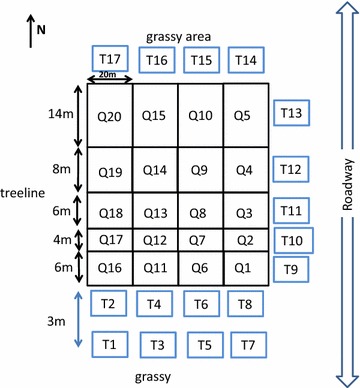
Schematic of the field showing layout of the blue sticky traps (*T*) and location of the quadrats (*Q*) planted with cabbage. Field site located near Brookfield, Prince Edward Island. Schematic not to scale

This one-field study, located in North Wiltshire (Latitude 46.3260379 Longitude −63.2825999), measured 400 × 40 m. The eastern end of the field measuring 80 m × 40 m was used for the study (Fig. 8). The field was surrounded on the north and south sides by grass and weed species, to the east by a roadway and to the west by the rest of the field (also planted in cabbage). The west side of the field was bordered by a brook and tree hedgerow. On the north, south and east sides, across the grassy areas and/or roadway were other fields used by this commercial grower. In 2013, the study field was planted in fall rye, while fields to the north and south were planted in lettuce. The field to the east was also planted in fall rye in 2013. In 2014 the study field was planted with cabbage, fields to the north, east and south were planted with fall rye.

Table 4 details the study plot. Twenty quadrats were established within the study plot. The two main varieties, Bronco and Lennox, were planted in 16 of the 20 quadrats, with approximately 100 heads/quadrat. The last four quadrats (5, 10, 15 and 20), planted with six other varieties, were not examined in enough replication to allow for statistical analysis. Cabbage plantings occurred as would be typical for a commercial organic grower. Varieties were planted sequentially from May 9 through June 17 with some varieties having multiple plantings and others having only a single planting. Quadrats were monitored for thrips throughout the study from July 22–September 2 (see *Onion thrips survey*). Harvest dates were July 31 or September 2, depending upon variety. At harvest, four heads from within each quadrat were weighed and measured. Measurements were along the polar and equator orientations of the cabbage. Cabbage plants were dissected to evaluate thrips damage within the head. Any thrips damage observed on the outer leaves and on layers 1 through 4 inclusive was recorded. The field was managed for cabbageworm and fungal infection with sprays of Coragen on July 25 at a rate of 150 ml/acre and Bravo on July 31, August 14 and August 30 at a rate of 1.5 L/acre. Fertilizer (Inca) was applied concurrent with Bravo at a rate of 1 L/acre.

**Table 4:** Details of plot sizes, number of plants/plot, planting and harvest dates and cultivars established in the trial on PEI, 2014

Planting date	Harvest date	Plot size/m	Plants/plot	Variety(s)	Quadrat(s)
May 9	July 31	20 × 6	396	Bronco	1, 6, 11, 16
May 15	July 31	20 × 4	264	Bronco	2, 7, 12, 17
May 15	Sept. 2	20 × 6	396	Lennox	3, 8, 13, 18
May 29	Sept. 2	20 × 8	528	Lennox	4, 9, 14, 19
June 9	July 31	20 × 3.5	231	Tiara, CLX	5, 10
June 9	Sept. 2	20 × 3.5	231	Grand Vantage, Bronco, Prime Vantage/Grand Vantage, Lennox	5, 10, 15, 20
June 17	July 31	20 × 3.5	231	BC63	10
June 17	Sept. 2	20 × 3.5	231	Bruno/Grand Vantage, Excalibur, Lennox	5, 10, 15 and 20

### Onion thrips monitoring

Thrips were surveyed in two ways: blue sticky traps and visual surveys. Traps were placed around the outside perimeter to monitor the thrips population migrating into the field. Visual surveys were used to quantify thrips numbers inside the plots as these would represent actual attack of the cabbage heads. Blue sticky traps measuring 10.2 cm × 12.7 cm from Solida (Montreal, Quebec) were clamped onto wooden slats and positioned approximately 60 cm above the ground. This height put them just above the developing cabbage head and foliage. Traps were established in the field on July 16, 2014 as the heads were beginning to form, consistent with the movement of thrips into the crop. Traps were placed along three sides of the field margin (North, East and South) outside each quadrat (see Fig. 8) with a second line of traps placed 3 m farther south of the southern edge. The second line was to determine if trapping could occur farther away from the field edge thereby avoiding damage from machinery. All traps were monitored twice each week beginning July 22 through to harvest in early September. Traps were changed each time and thrips identified in the laboratory using a microscope. On the same days as the traps were monitored, four cabbage heads from within each quadrat were surveyed for presence of thrips by visual surveying of the outer layer and under the 1st leaf layer of the cabbage head.

### Statistical analysis

All analyses were done using unbalanced ANOVA in R unless indicated otherwise (R-Core Team 2012). To examine the thrips population around the study site over time, thrips captured on sticky traps were pooled across location and compared across capture dates. The effect of trap location was evaluated by pooling traps within each location (i.e. north, east, south-edge and south-outer) and comparing across capture dates. Thrips entering the field site were compared with thrips within the field site by correlating cabbage head thrips counts with trap catches throughout the season by date. The influence of variety and planting date on total thrips observed on cabbage heads over the growing season was analysed. Location of thrips within the cabbage head at harvest, cabbage head weight and cabbage size was analysed for the two main varieties, Bronco and Lennox, and planting date. Percentage of cabbage heads showing thrips damage were analysed by location with the cabbage head and correlated with number of thrips present at harvest and the total thrips present on the heads over the season. And finally, correlation between trap catches and head weight and cabbage size was examined using linear models. Cabbage head weight and size (circumference along polar and equator dimensions) were compared for variety, planting date and harvest date. Thrips counts were transformed by sin^−1^(sqrt(x + 0.5)) where necessary prior to ANOVA where homogeneity of variance was violated.
